# Case Report: Use of mirvetuximab soravtansine in a patient with platinum-resistant ovarian cancer and concomitant PARP-inhibitor-related myelodysplastic syndrome

**DOI:** 10.3389/fonc.2026.1785547

**Published:** 2026-04-20

**Authors:** Sophonie Shilonite Njonou Noujiep, Judith Altmann, Lars Bullinger, Jalid Sehouli

**Affiliations:** 1 Department of Gynecology with Center of Oncological Surgery, Charité – Universitätsmedizin Berlin, Corporate Member of Freie Universität Berlin and Humboldt-Universität zu Berlin, Berlin, Germany; 2 Department of Hematology, Oncology and Cancer Immunology, Charité – Universitätsmedizin Berlin, Corporate Member of Freie Universität Berlin and Humboldt-Universität zu Berlin, Berlin, Germany; 3 German Cancer Consortium (Deutsches Konsortium Für Translationale Krebsforschung, DKTK), Berlin, Germany; 4 National Center for Tumor Diseases (NCT), Berlin, Germany

**Keywords:** myelodysplastic syndrome (MDS), PARP inhibitor (PARPi), ovarian cancer, mirvetuximab soravtansine (MIRV), antibody-drug conjugate (ADC)

## Abstract

Response rates in platinum-resistant ovarian cancer remain low (16–30%) and decline with subsequent lines of therapy. Mirvetuximab soravtansine (MIRV), an antibody–drug conjugate targeting folate receptor alpha (FRα), has demonstrated clinically meaningful activity in FRα-positive disease. We report a 65-year-old patient with heavily pretreated, FRα-positive ovarian cancer who developed therapy-related myelodysplastic syndrome with increased blasts (MDS-IB2, DNMT3A-mutated) during therapy with MIRV in combination with carboplatin having received prior PARPi maintenance therapy. Azacitidine treatment induced complete hematologic remission. Following progression of the ovarian cancer disease, MIRV was reintroduced concurrently with ongoing azacitidine. This strategy resulted in seven months of sustained disease control of the ovarian cancer without evidence of MDS worsening. In fact, after three cycles of azacitidine, a follow-up bone marrow biopsy showed no residual MDS. This case demonstrates that MIRV can be safely and effectively administered alongside azacitidine, providing clinically meaningful tumor control without compromising hematologic outcomes. These findings support the concurrent management of ovarian cancer and therapy-related MDS as a viable and underutilized treatment approach in a highly challenging clinical setting.

## Introduction

Platinum-resistant ovarian cancer is associated with limited treatment options and overall response rates of 16–30%, which decline sharply with third- or fourth-line therapy (1). Current strategies rely on non-platinum cytotoxic agents, including liposomal doxorubicin, etoposide, and gemcitabine, often combined with bevacizumab (2); however, outcomes remain suboptimal.

Mirvetuximab soravtansine (MIRV) is an antibody–drug conjugate (ADC) targeting folate receptor alpha (FRα), conjugated to the cytotoxic maytansinoid DM4 (1, 2). By selectively binding FRα on tumor cells, MIRV delivers DM4 intracellularly, inducing apoptosis via anti-microtubule activity while sparing most normal tissues. FRα is highly expressed in ovarian cancer, with reported prevalence of 76–89%, supporting the rationale for targeted therapy (1). MIRV has demonstrated meaningful clinical activity in heavily pretreated, FRα-positive ovarian cancer, with a favorable safety and hematologic profile relative to conventional chemotherapy.

**Figure 1:**
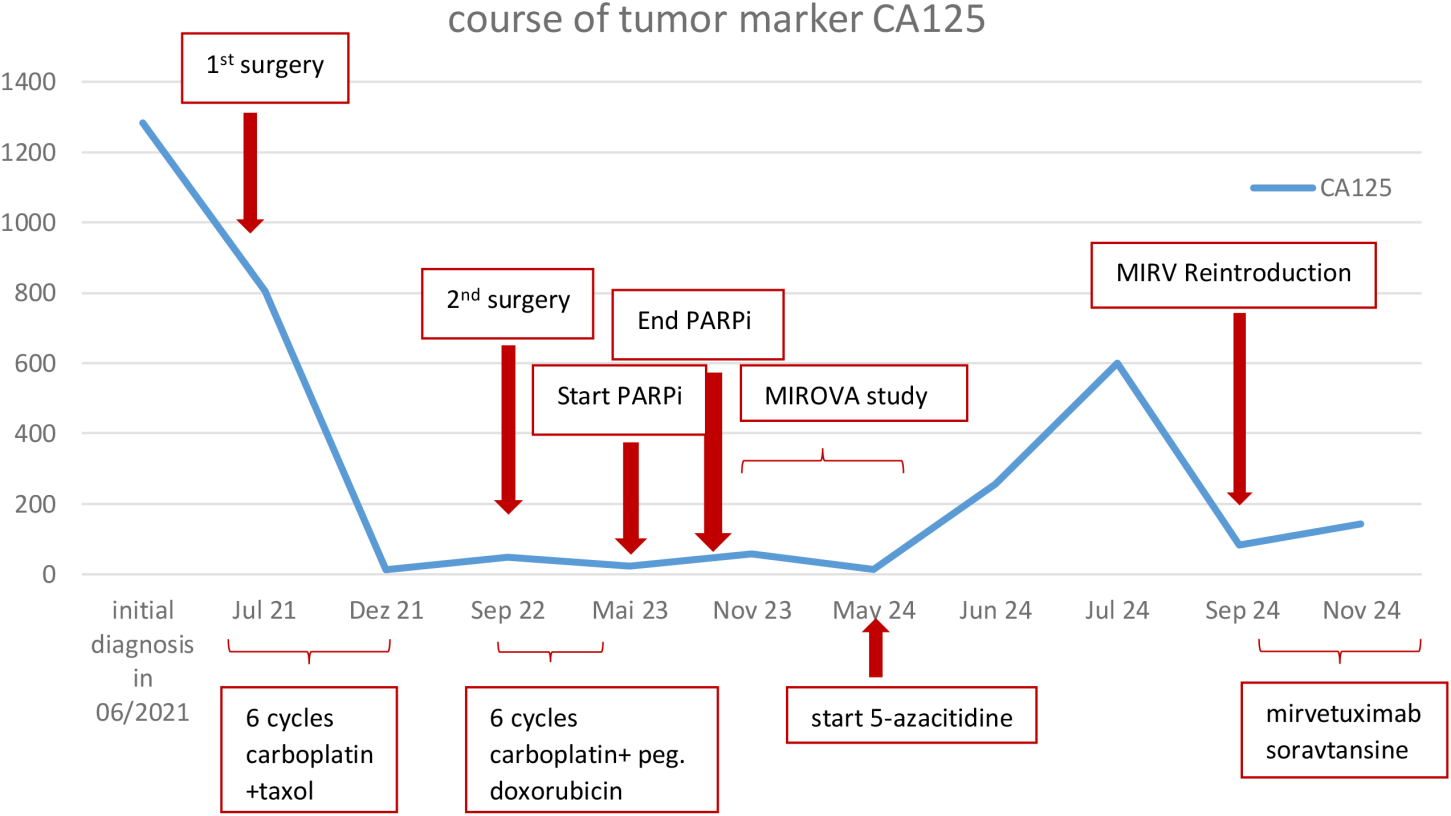
Development of the serological marker CA 125. Integrated clinical timeline.

We report the case of a 65-year-old patient with platinum-resistant, FRα-positive ovarian cancer complicated by PARP inhibitor–associated myelodysplastic syndrome (MDS), who was successfully managed with concurrent azacitidine and MIRV, achieving sustained tumor control without hematologic compromise (**Figure 1**).

## Case description and diagnostic assessment

A 65-year-old woman was diagnosed in June 2021 with advanced high-grade serous ovarian carcinoma presenting with malignant ascites and peritoneal carcinomatosis (FIGO IIIC). The family history for cancer was positive, with the patient`s sister having ovarian cancer, her father having colon cancer, and a grand-aunty on the maternal side having breast cancer.

Primary cytoreductive surgery achieved no residual disease, followed by standard platinum-based chemotherapy (six cycles) with bevacizumab. Molecular testing was negative for BRCA mutations and homologous recombination deficiency.

The patient experienced two subsequent recurrences over the following two years, managed with recurrence surgery, platinum-based chemotherapy, and maintenance therapy with the PARP inhibitor niraparib between both recurrences. During PARP inhibitor treatment, the disease progressed, and the patient was enrolled in the MIROVA trial (ClinicalTrials.gov ID: NCT04274426), receiving mirvetuximab soravtansine (MIRV) 6 mg/kg in combination with carboplatin AUC 5.

**Figure 2:**
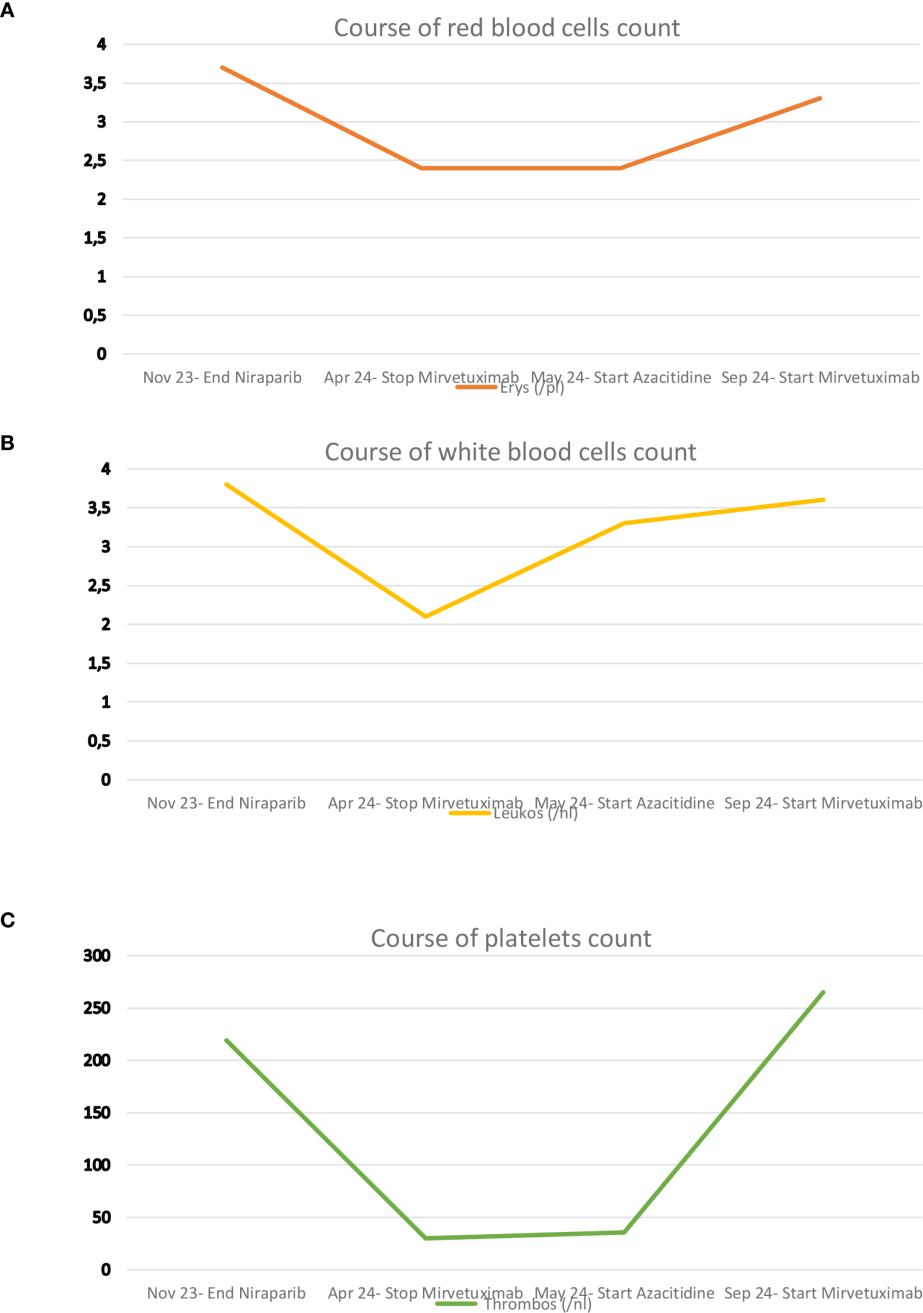
**(A–C)** show the development of red blood cell count, white blood cell count, and platelet count, respectively.

After four cycles, in April 2024, persistent pancytopenia was observed. Findings included hemoglobin 8.4 g/dL, erythrocytes 2.3/pL, leukocytes 1.6/nL, and platelets 25/nL as shown in **Figure 2**. This led to bone marrow evaluation, confirming therapy-related myelodysplastic syndrome with increased blasts (MDS-IB2) and a DNMT3A mutation. Azacitidine therapy (75 mg/m²/day subcutaneously for seven consecutive days every 3 weeks) induced complete hematologic remission within three cycles.

Following subsequent progression of ovarian cancer in August 2024, MIRV was reintroduced concurrently with ongoing azacitidine. At this time, hematologic remission of MDS was still observed, and therapy with Azacitidine was ongoing. This combined approach resulted in sustained disease control of ovarian cancer for seven months, accompanied by a marked decline in CA-125 and stable blood counts (hemoglobin 11.3 g/dL, erythrocytes 3.7/pL, leukocytes 4.4/nL, platelets 180/nL), without evidence of MDS exacerbation.

Disease progression ultimately occurred with hepatic and peritoneal metastases. Biopsy of the liver lesion confirmed high-grade serous adenocarcinoma with HER2 score 1+ and TROP2 expression of 90%. Considering the patient’s overall condition and MDS, the following therapeutic options were discussed: 1. Platinum re-induction monotherapy at a potentially reduced dose (weekly or three-weekly); 2. Gemcitabine monotherapy; 3. Off-label deruxtecan based on HER2 1+ positivity (Pan-DESTINY study); 4. Sacituzumab govitecan based on TROP2 expression.

Given extensive prior therapy and clinical status, coverage approval for deruxtecan monotherapy was requested. Surgery was not indicated due to extra-abdominal multifocal tumor formation and prior multivisceral surgery (2021). Platinum re-induction was considered high-risk given fragile bone marrow and prior PARP inhibitor exposure. Achieving remission was deemed to offer limited benefit for maintenance therapy. Gemcitabine at 800–1000 mg/m², initially at a dosage of 800 mg/m², was recommended.

Because further treatment options were limited by prior therapies and bone marrow vulnerability, the patient was transitioned to palliative management, with consideration of antibody–drug conjugates as subsequent therapy.

This clinical course highlights the development of therapy-related MDS under PARP inhibition, its successful reversal with azacitidine, and—most notably—the safe and effective reintroduction of MIRV under ongoing hypomethylating therapy. The observed seven-month disease control without hematologic compromise underscores the feasibility and clinical relevance of this concurrent treatment strategy in a highly challenging setting.

## Discussion

Mirvetuximab soravtansine (MIRV) represents a significant advancement for platinum-resistant, FRα-positive ovarian cancer (3). Its antibody–drug conjugate (ADC) design enables selective delivery of the cytotoxic payload DM4 to FRα-expressing tumor cells, limiting systemic exposure and reducing myelosuppression relative to conventional chemotherapy. Clinical data from the FORWARD I and SORAYA trials support this profile, demonstrating lower rates of grade ≥3 hematologic toxicity even in heavily pretreated populations (3, 4). In the present case, MIRV was safely reintroduced concurrently with azacitidine in a patient with therapy-related myelodysplastic syndrome (MDS), achieving seven months of disease control without compromising hematologic recovery. At the time of ovarian cancer progression, MDS was in complete remission under ongoing azacitidine, highlighting the tolerability and feasibility of this combined approach.

MDS is an umbrella term for a heterogeneous group of acquired clonal disorders of the hematopoietic stem cells that leads to bone marrow insufficiency and cytopenia with an increased risk of progression into an acute myeloid leukemia (AML) (5). The prevalence of MDS increases with age, with a median age of onset over 70 years. While approximately 90% of cases are idiopathic, secondary forms of MDS develop due to damage to hematopoietic progenitor cells (blasts) following chemotherapy, radiation therapy, radioiodine therapy or, as in our case, PARPi treatment (6, 7).

While main adverse events related to PARPi are usually transient hematological changes including thrombocytopenia, anemia, and neutropenia occurring during the first three months of treatment, MDS is a feared delayed treatment-related adverse event of PARPi with potentially life-threatening implications. In a safety meta-analysis published in 2021 28 randomized-controlled trials including 5693 patients in PARPi groups and 3406 patients in control groups were analyzed (6). Based on the 18 placebo RCTs, PARPi significantly increased the risk of MDS and AML compared with placebo treatment (2.63 [95% CI 1.13–6.14], p=0.026) (6). The incidence of MDS and AML across PARPi groups was 0.73% (95% CI 0.50–1.07) and 0.47% (0.26–0.85) across placebo groups (6).

Median duration of PARPi exposure was 9.8 months, ranging from 0.2 to 66.8 months. The median latency period of MDS and AML from first exposure to a PARPi was 17.8 months (range: 0·6 to 66·8 months) (6). MDS occurred after a median of 17.8 months from first PARP inhibitor exposure, and AML after 20.6 months. In this cohort, AML was reported as progressing from MDS in 8% of patients (6). Associated symptoms were most frequently anemia, followed by thrombocytopenia, neutropenia, and pancytopenia (6). Complete blood count monitoring is recommended before and monthly after starting a PARPi. Patients who have not recovered within 28 days or have persistent cytopenia following dose modification, further investigation including bone marrow analysis and blood sample for cytogenetics must be done additionally. PARPi must be discontinued if MDS or AML are confirmed (6).

In the above described case, MDS was detected 11 months after the start of PARPi treatment with niraparib, which was only given for 5 months and paused due to a second relapse of the ovarian cancer. The etiology of MDS in this patient is likely multifactorial. PARP inhibitors (PARPi), including niraparib, are associated with delayed hematologic toxicity, including MDS and acute myeloid leukemia. However, prior cytotoxic therapies, particularly platinum-based regimens such as carboplatin, are well-documented contributors to therapy-related MDS. In addition, the presence of a DNMT3A mutation raises the possibility of pre-existing clonal hematopoiesis (CHIP), which can facilitate clonal evolution under genotoxic stress. Thus, while PARPi exposure temporally coincided with MDS onset, cumulative prior therapy and CHIP may have contributed, underscoring the need to interpret PARPi-associated MDS in the context of the full treatment history. Real-world analyses confirm that PARPi-associated MDS/AML is rare but clinically significant, often developing in patients with extensive prior therapy, supporting the relevance of this case.

This report also highlights the feasibility and clinical significance of combining MIRV with azacitidine. The patient tolerated the combination well, with no exacerbation of MDS, stable blood counts, and meaningful tumor control for seven months. This is particularly relevant for patients in whom standard cytotoxic chemotherapy is contraindicated due to impaired marrow reserve. ADC therapy thus offers a targeted strategy to maintain anti-tumor efficacy while minimizing hematologic toxicity.

Overall, this case illustrates the complexity of managing recurrent ovarian cancer in patients with therapy-related MDS. It emphasizes careful assessment of contributing factors, including prior platinum exposure and pre-existing CHIP, and provides evidence that MIRV can be safely integrated with hypomethylating therapy. As PARPi use expands in clinical practice, such cases are expected to become more frequent, highlighting the need for evidence-based approaches to concurrently manage ovarian cancer and therapy-related hematologic disorders.

Written informed consent for publication was obtained from the patient.

## Data Availability

The original contributions presented in the study are included in the article/supplementary material. Further inquiries can be directed to the corresponding author.
